# Evidence for a Role of the Polysaccharide Capsule Transport Proteins in Pertussis Pathogenesis

**DOI:** 10.1371/journal.pone.0115243

**Published:** 2014-12-12

**Authors:** Regina Hoo, Jian Hang Lam, Ludovic Huot, Aakanksha Pant, Rui Li, David Hot, Sylvie Alonso

**Affiliations:** 1 Department of Microbiology, Immunology Programme, Yong Loo Lin School of Medicine, National University of Singapore, Centre for Life Science #03-05, 28 Medical Drive, Singapore 117597, Singapore; 2 Transcriptomics and Applied Genomics, Institut Pasteur de Lille, Centre for Infection and Immunity of Lille (CIIL), U1019, UMR8204, 1 rue du Professeur Calmette, F-59019 Lille, France,; Universidad Nacional de La Plata., Argentina

## Abstract

Polysaccharide (PS) capsules are important virulence determinants for many bacterial pathogens. *Bordetella pertussis*, the agent of whooping cough, produces a surface associated microcapsule but its role in pertussis pathogenesis remained unknown. Here we showed that the *B. pertussis* capsule locus is expressed *in vivo* in murine lungs and that absence of the membrane-associated protein KpsT, involved in the transport of the PS polymers across the envelope, but not the surface-exposed PS capsule itself, affects drastically *B. pertussis* colonization efficacy in mice. Microarray analysis revealed that absence of KpsT in *B. pertussis* resulted in global down-regulation of gene expression including key virulence genes regulated by BvgA/S, the master two-component system. Using a BvgS phase-locked mutant, we demonstrated a functional link between KpsT and BvgA/S-mediated signal transduction. Whereas pull-down assays do not support physical interaction between BvgS sensor and any of the capsule locus encoded proteins, absence of KpsT impaired BvgS oligomerization, necessary for BvgS function. Furthermore, complementation studies indicated that instead of KpsT alone, the entire PS capsule transport machinery spanning the cell envelope likely plays a role in BvgS-mediated signal transduction. Our work thus provides the first experimental evidence of a role for a virulence-repressed gene in pertussis pathogenesis.

## Introduction

PS capsules represent the outermost structure of some bacteria species and play an important role in protecting them from unfavorable or hostile environments. Apart from acting as a protective physical barrier, bacterial capsules have been recognized as an important virulence determinant by mediating host-pathogen interactions and evasion from host immune responses, including resistance to antimicrobial peptides [1], inhibition of neutrophil recruitment [2], resistance to phagocytosis [3], [4] and resistance to complement killing [5]. Capsules have also been associated with the later developmental stages of complex biofilm structures that display enhanced resistance to antibiotics [6].

The Gram-negative bacterium *Bordetella pertussis* is the causative agent of pertussis or whooping cough. According to World Health Organization statistics in 2010, pertussis is one of the ten most common causes of death from infectious disease worldwide, accounting for 300,000–400,000 deaths each year. The introduction and global implementation of pertussis vaccination over the past 60 years have successfully reduced the mortality and incidence rate of pertussis among young children. However, cases of pertussis infections in adult have been increasingly reported [7]–[9], suggesting that current pertussis vaccination strategies must be improved and prompting the development of new pertussis vaccine candidates [10]. *B. pertussis* produces a variety of virulence factors including toxins, adhesins and many others which are regulated by the BvgA/S two-component system in response to environmental stimuli. BvgA/S activation is characterized by a sophisticated His-Asp-His-Asp phosphorelay transfer mechanism from the integral inner membrane spanning sensor; BvgS to the cytoplasmic transcriptional activator; BvgA [11], [12]. Under virulent or Bvg^+^ phase *in vitro* culture conditions, phosphorylated BvgA (P-BvgA) displays an increased affinity for *bvg*-activated promoters, leading to the up-regulation of more than a hundred genes involved in virulence, and referred to as *bvg*-activated genes (*vag*). Also, in Bvg^+^ phase, transcription of another set of genes known as *bvg*-repressed genes (*vrg*) is repressed by the *vag*-encoded transcriptional repressor protein, BvgR [13]. In contrast, the avirulent or Bvg^-^ phase in the presence of MgSO_4_ or nicotinic acid, is characterized by minimal expression of *vags* and maximal expression of *vrgs*. An intermediate phase, namely Bvg^i^ has more recently been described [14], [15] The role of the *vrg*-encoded products family in *B. pertussis* virulence has not been clearly established and remains to be demonstrated [16].

Recently, we reported that *B. pertussis* produces an intact PS microcapsule at its bacterial surface [17], [18]. The *B. pertussis* capsule locus is organized in a 10kb-operon, which comprises genes involved in transport, biosynthesis and modification/export of a putative type II PS [19]. The *B. pertussis* capsule operon belongs to the *vrg* family with maximal expression under *in vitro* Bvg^-^ phase and basal expression in Bvg^+^ phase [17]. We showed that the *B. pertussis* PS capsule is not involved in classical capsule-mediated defense mechanisms, including adherence to mammalian host cell, complement-mediated killing and antimicrobial attack [17]. Currently, it is not known whether the *B. pertussis* PS capsule plays any role in bacterial virulence within an infected host.

In this study, we characterized the expression and the role of the capsule locus in pertussis pathogenesis. We showed that, KpsT, a membrane associated protein involved in the transport of the PS capsule across the cell envelope is necessary for optimal BvgA/S-mediated signal transduction. Our data support a structural role of KpsT and possibly the entire PS capsule transport machinery in the bacterial cell membrane integrity, which consequently impacted on the BvgA/S-mediated virulence gene regulation.

## Materials and Methods

### Bacterial strains and growth conditions

All *B. pertussis* and isogenic mutant derivatives strains used in this study are listed in Table 1. All *B. pertussis* strains were grown at 37°C on Bordet-Gengou (BG) agar (Difco) supplemented with 10% defibrinated sheep blood with 1% glycerol or in modified Stainer-Scholte (SS) medium containing 2,6-O-dimethyl-β-cyclodextrin (Sigma Chemical) at 1 g/liter supplemented with either 10 µg/ml gentamicin, 100 µg/ml streptomycin or 30 µg/ml chloramphenicol. All DNA manipulations in *E. coli* were carried out using chemically competent *E. coli* One-Shot TOP10 (Invitrogen). *E. coli* strains were grown at 37°C overnight in fresh Luria-Bertani broth or on LB agar (Difco) plates. When appropriate, 100 µg/ml ampicillin, 50 µg/ml kanamycin, 10 µg/ml gentamicin, 30 µg/ml chloramphenicol was added to select for antibiotic-resistant strains.

**Table 1:** *B. pertussis* strains used in this study.

B. pertussis strains	Genotype/Relevant features	Source
BPSM	Tohama I derivative, mutant rpsL	[72]
ΔkpsT	BPSM carrying an in-frame deletion in kpsT ORF	This study
ΔkspE	BPSM carrying an in-frame deletion in kpsE ORF	This study
ΔvipC	BPSM carrying an in-frame deletion in vipC ORF	This study
KOcaps	BPSM carrying an in-frame deletion from kpsM to wcbO ORFs	[17]
ΔkpsTcom	BPSM carrying an in-frame deletion in kpsT ORF containing vector pBBR::Pcaps-kpsT	This study
BvgS-VFT2	BPSM carrying amino acid substitution at F375E and Q461E at the periplasmic VFT2 domain	[31]
BvgS-VFT2-ΔkpsT	BvgS-VFT2 carrying an in-frame deletion in kpsT ORF	This study
KOcaps:kpsT	KOcaps containing vector pBBR::Pcaps-kpsT	This study
KOcaps:kpsMT	KOcaps containing vector pBBR::Pcaps-kpsMT	This study
BPSH	BPSM derivative expressing his-tagged BvgS at the N-terminal	This study
BPSH-KOcaps	BPSH carrying an in-frame deletion from kpsM to wcbO ORFs	This study
BPSH-ΔkpsT	BPSH carrying an in-frame deletion in kpsT ORF	This study
BPSH-ΔkpsTcom	BPSH carrying an in-frame deletion in kpsT ORF containing vector pBBR::Pcaps-kpsT	This study

### Construction of *B. pertussis* capsule-deficient mutant strains

Non-polar single gene deletion was constructed for *kpsT*, *kpsE* and *vipC* ORFs in wild-type BPSM via double homologous recombination method. Briefly, approx. 600–800 bp of genes, termed as PCR1, flanking from the 5′ internal region and PCR2, flanking from the 3′ internal region of the respective ORFs to be deleted, were first amplified from wilt-type BPSM gDNA using the oligonucleotides stated in Table 2, inserted into pCR2.1-TOPO and verified by DNA sequencing. The PCR1 and PCR2 fragments were then sequentially digested with restriction enzymes from TOPO vector, inserted into the intermediate vector pBR322 and finally into the *Bordetella* suicide vector pJQ200mp18rpsl, yielding pJQT1-2, pJQE1-2 and pJQV1-2 respectively. The recombinant pJQ constructs were used for allelic exchange in wild-type BPSM, yielding Δ*kpsT*, Δ*kpsE* and Δ*vipC* mutant strain respectively as described previously [20]. pJQT1-2 was used for allelic exchange in BvgS-VFT2 strain yielding BvgS-VFT2-Δ*kpsT* mutant strain.

**Table 2:** List of forward and reverse primers used in cloning.

Oligo	Sequence (5′ to 3′)	Description
kpsM1F	ttggatcctgtccaccaccatctacgtggtgt	Forward primer to amplify PCR1-*kpsT*
kpsM2R	ttgctagccagctccatgccgcagatca	Reverse primer to amplify PCR1-*kpsT*
kpsE1F	ttgctagccttggacgaaaccatcgcgc	Forward primer to amplify PCR2-*kpsT*
kpsE2R	ttaagcttgccagctgcagattggcctc	Reverse primer to amplify PCR2-*kpsT*
kpsT1F	ttgaattccgcatgatctgcggcatcga	Forward primer to amplify PCR1-*kpsE*
kpsT2R	ttaagcttgacatactggtcggacgcaat	Reverse primer to amplify PCR1-*kpsE*
wbpT7F	ttaagcttgaggccaatctgcagctggc	Forward primer to amplify PCR2-*kpsE*
wbpT6R	ttggatcctatgcccgcggcgcggctt	Reverse primer to amplify PCR2-*kpsE*
wbpTF	ttgaattccatgccgccggtggaccg	Forward primer to amplify PCR1-*vipC*
wbpTR	ttaagcttacggcacatgcccagcacg	Reverse primer to amplify PCR1-*vipC*
wzaF	ttaagcttgagttcgagccggtgctgg	Forward primer to amplify PCR2-*vipC*
wzaR	ttggatccttgctggtaaggaatgcgctg	Reverse primer to amplify PCR2-*vipC*
kpsTcomF	ttggatcccgttgatggagacggccatg	Forward primer to amplify full length *kpsT*
kpsTcomR	ttaagctttcaggattgctcagcgtcgac	Reverse primer to amplify full length *kpsT*
BvgA-BamHI-F	ttggatcctgtactgagattcgccgtc	Forward and reverse primer to amplify PCR1 from 3′ end of *bvgA* ORF to 5′ end of *bvgS* signal peptide ORF
BvgS-XbaI-R	tttctagagcttgcctgcgcgggc	
BvgS-XbaI-6His-F	tttctagacatcatcaccatcaccaccaggagctgaccctg	Forward and reverse primer to amplify PCR2 downstream of *bvgS* signal peptide sequence; forward primer carries nucleotides encoding 6x histidines
BvgS-HindIII-R	ttaagcttggcgactacgcgaacgtcattgaa	

Restriction sites are underlined.

### Construction of Δ*kpsT* complemented strain

Full length *kpsT* ORF was amplified with primers kpsTcomF and kpsTcomR listed in Table 2, cloned into TOPO vector and verified by DNA sequencing. pUC57-Pcaps containing the 866 bp BPSM native capsule promoter (Genescript) was cloned into *Xba*I-*BamH*I-opened pBBR1MCS [21], followed by ligation with the *kpsT* ORF, yielding pBBR::Pcaps*kpsT*. The recombinant pBBR::Pcaps*kpsT* plasmid was electroporated into Δ*kpsT* mutant strain, yielding the complemented strain, designated as Δ*kpsT*com.

### Construction of recombinant *B. pertussis* BPSH strain

To construct *B. pertussis* BPSH strain expressing histidine tag at the N-terminal end of BvgS, PCR1 fragment was amplified from 3′ end of *bvgA* ORF to the end of *bvgS* signal peptide ORF whereas PCR2 fragment carrying six histidines encoding sequences from primer overhang was amplified from downstream of *bvgS* signal peptide using oligonuclotides listed in Table 2. The PCR1 and PCR2 fragments were inserted into pCR2.1-TOPO and verified by DNA sequencing. PCR1 and PCR2 were sequentially digested with restriction enzymes from TOPO vector and finally into the *Bordetella* suicide vector pJQ200mp18rpsl, yielding pJQ-His-BvgS. pJQ-His-BvgS were inserted into the *bvgS* chromosomal locus of wild-type BPSM via allelic exchange, yielding BPSH stain. pJQ-KO*caps* [17] and pJQT1-2 was used for allelic exchange in BPSH, yielding BPSH-KO*caps* and BPSH-as Δ*kpsT* respectively. pBBR::Pcaps*kpsT* plasmid was electroporated into BPSH-Δ*kpsT* mutant strain, yielding the complemented strain, designated as BPSH-Δ*kpsT*com.

### Southern blot analysis

Chromosomal DNA was extracted and purified from BPSM and Δ*kpsT* and Δ*kpsE* bacteria using Genomic-tip 100/G Anion-Exchange Resin (Qiagen) and Genomic DNA Buffer Set (Qiagen) according to the manufacturer's instructions. 1 µg of chromosomal DNA from *B. pertussis* strains were digested with restriction enzymes for 4 hrs and subjected to 0.8% agarose gel electrophoresis. The agarose gel containing the digested DNA was chemically treated and transferred onto a nitrocellulose membrane (Milipore) according to Roche's DIG application manual. The membrane was UV-fixed for 1 min and equilibrated with 10 ml pre-heated DIG Easy Hyb solution (Roche) at 65°C for 20 min, with gentle agitation. A digoxigenin (DIG)-labelled probe was amplified using the PCR DIG Probe Synthesis Kit (Roche) according to the manufacturer's instructions. For hybridization, about 5-25 ng/ml of heat-denatured DIG-labeled DNA probe in DIG Easy Hyb solution was incubated with the membrane overnight at 65°C. Detection was performed using alkaline phosphatase-conjugated anti-DIG antibody (Roche) at a dilution of 1∶5,000. The membrane was developed using NBT/BCIP AP substrate (Chemicon).

### Whole cell extract and supernatant concentration preparation

Mid-exponential virulent phase bacteria grown in 10 ml of SSAB medium were harvested at equal OD_600 nm_ and prepared as described previously [22]. For purification of His-BvgS, whole cell extract was harvested from mid-exponential virulent phase bacteria grown in 50 ml of SSAB medium were harvested at equal OD_600 nm_ and washed twice in 25 ml of 1x PBS. The washed bacteria pellet was resuspended in 5 ml of lysis buffer A (20 mM Tris-HCl pH 7.9, 10 mg/ml lysozyme, 50 µM KCL, 10% glycerol and 1x protease inhibitor) and the bacteria were incubated at 37°C with rocking for 1 hr and broken in a bioruptor for 15 min. Cellular contents and debris were removed after centrifugation at 8000 rpm for 10 min. The pellet was resuspended again in 5 ml lysis buffer A with addition of 1% Triton-X-100 and bacteria were incubated at 37°C with rocking for 1 hr, prior to centrifugation at 10,000 rpm for 10 min. Finally, the bacteria pellet was solubilized in 5 ml solubilization buffer (20 mM Tris-HCl, 50 µM KCL, 10% glycerol and 6 M guanidine hydrochloride at final pH 8) at 4°C with rocking for 1 hr. The cell lysate was clarified by centrifugation at maximum speed for 15 min to pellet unsolubilized cells and debris.

### Purification of His-BvgS by affinity chromatography

The clarified lysate was first measured for its protein concentration using BCA Protein Assay Kit (Thermo). Approximately 5 mg of total protein lysate with a final concentration of 20 µM imidazole was mixed with 400 µl of Ni-NTA slurry (Qiagen). Overnight binding was performed at 4°C with gentle rocking, thereafter the cellular lysate was loaded onto an empty chromatography column for gravity flow purification. The column was washed with 5 column volume of wash buffer (6 M urea, 100 mM NaH_2_PO_4_, 10 mM Tris-Cl and 20 µM of imidazole pH 6.3). His-BvgS protein was batch eluted four times in elution buffer (6 M urea, 100 mM NaH_2_PO_4_, 10mM Tris-Cl and 200 µM of imidazole pH 4.5).

### SDS-PAGE and Western blot analysis

Whole cell extract and 10x concentrated supernatant of *B. pertussis* cultures were run on 8% or 12% SDS-PAGE as previously described [22]. The transferred PVDF membranes were incubated with mouse anti-FHA monoclonal antibody (National Institute for Biological Standards and Control, UK) diluted 1∶2000, mouse anti-PT monoclonal antibody diluted 1∶1,500 (National Institute for Biological Standards and Control, UK), rabbit anti-BrkA polyclonal antibodies diluted 1∶30,000 (custom-made, New England Peptide, Gardner, MA). The membrane was then incubated with appropriate AP-conjugated goat anti-mouse or anti-rabbit IgG secondary antibodies (Bio-Rad), both diluted 1∶3,000, and revealed by chromogenic detection after addition of NBT/BCIP AP substrate. Purified His-BvgS were run on 10% SDS-PAGE and were either stained with Coomassie blue or transferred onto PVDF membranes, incubated with rat anti-BvgS polyclonal antibodies (kind gift from Dr. F. J. Dubuisson) diluted 1∶3000 and mouse anti-penta His-HRP conjugated monoclonal antibodies (Qiagen) diluted 1∶10,000 in blocking solution for 1 hr at room temperature or overnight in 4°C. The membrane incubated probed with anti-BvgS was incubated with goat anti-rat IgG secondary antibody diluted 1∶5000 (Cell Signaling), and revealed by substrate for chemiluminescent detection (GE Healthcare).

### RNA extraction from *in vitro B. pertussis* culture

0.5 ml of *B. pertussis* bacteria in virulent phase SSAB medium were harvested at OD_600 nm_ 2 and incubated with RNAprotect Bacteria reagent (Qiagen) for RNA stabilization. The pelleted bacteria were then resuspended in 100 µl of Tris-EDTA (TE) containing 20 mg/ml lysozyme and incubated at room temperature for 20 min. RNA extraction was then performed using RNeasy Minikit (Qiagen) for RT-PCR and real-time PCR reactions, or TirReagent (Ambion) for microarray analysis, according to the manufacturer's instructions. Purified RNA was treated using the RNase free-DNAse set (Qiagen) to remove contaminant DNA. Reverse transcription was performed on 10 ng of bacterial RNA using the iScript cDNA synthesis kit (Bio-Rad).

### RNA extraction from *B. pertussis* infected mice lungs

*B. pertussis*-infected BALB/c mice were euthanized and their lungs were aseptically removed and immediately immersed in 3 ml of RNAprotect Bacteria Reagent (Qiagen) for 1 hr in 4°C. The stabilized lungs were homogenized using the High Shear homogenizer (Omni International, Reasearch Biolabs). The lung homogenates from individual lungs were filtered through a cell strainer. The filtered suspension was centrifuged at 1,500 rpm for 7 min to pellet the remaining cell debris. The supernatant containing free bacteria was centrifuged at 10,000 rpm for 10 min. The bacterial pellet was again stabilized in 1 ml of RNAprotect Bacteria Reagent for 5 min at room temperature. Bacterial RNA was extracted using lysozyme and RNeasy Mini Kit buffer as described above. In-solution DNA digestion was performed with DNase-I, and finally total RNA was eluted in 20-30 µl of DPEC water.

### Real-time polymerase chain reaction

Real-time PCR was performed in a 96 well-plate with each well containing 2 µl of cDNA mix, 0.5 µl of forward and reverse primers (0.5 µM final) listed in Table 3 and 25 µl SYBR-Green Supermix with ROX (Bio-Rad) to a final volume of 50 µl. Samples were run in triplicate. Real-time PCR amplification was conducted with the ABI Prism 7500 sequence detector (Applied Biosystems) over 35 cycles and with an annealing temperature of 63°C. Expression of each target gene was based on relative quantification using the comparative critical threshold (Ct) value method. Relative quantification of a specific gene was evaluated in each reaction by normalization to the Ct value obtained for the endogenous control gene, *recA* [19]. Control reactions without cDNA were used as negative controls.

**Table 3:** List of forward and reverse primers (from 5′ to 3′) used in Real-time PCR analysis.

Gene	Forward primer (5′-3′)	Reverse primer (5′-3′)
*bvgA*	TCCTCATCATTGACGATCACCC	CGATGACTTCCAGCCCGTCCA
*bvgR*	AACAGCTGCTGGCGCAGGTT	GCCGCAGGCTATGCAGGCTT
*brkA*	GTATCTCGATAGATTCCGTCAAT	CGTGTTGTCCCGTGGTCG
*fhaB*	TGTCCGCCATGGAGTATTTCAA	CCCAAATGTACTCGTAGCGATTC
*ptx*	GCGTTGCACTCGGGCAATTC	CAGATGGTCGAGCACATTGTC
*sphB1*	TGCTGCAGGACAACCTGTATTC	TCAGGCCGGCCGAGACTTCG
*recA*	GACGACAAAACCAGCAAGGCC	CGTAGACCTCGATCACGCGG

### Microarray production and analysis

Long oligonucleotide probes were designed on the sequences of the 3554 open reading frames (all coding CDS except transposases of IS elements) of *B. pertussis* Tohama I genome using OligoArray v2.1 [23]. Oligonucleotides were synthesized by Sigma-Aldrich and spotted on Nexterion AL slides (Schott Nexterion) in 1x SciSpot-AM buffer (Scienion) using a Q-Array II spotter (Genetix). For each sample, 15 µg of total RNA was reverse transcribed with 400 units of SuperScript III (Invitrogen) in presence of 100 µM Cy3-dCTP or Cy5-dCTP (GE) and 300 mM of hexanucleotide random (Roche). The labelled cDNA was NaOH treated to degrade RNA and purified on Qiaquick PCR purification kit (Qiagen). Hybridization was performed in 40% formamide, 5x Denhardt's solution, 0.1% SDS, 1 mM Sodium pyrophosphate and 5x SSC during 14–16 h at 52°C under agitation. Slides were then washed sequentially in 2× SSC/0.2% SDS during 5 min, 0.5× SSC during 10 min, 0.05x SSC during 5 min and 0.01x SSC during 1 min before drying. Hybridized slides were scanned using an Innoscan 700 (Innopsys) microarray scanner and analyzed with Mapix v3.1 (Innopsys). Normalisation and differential expression were carried out using the LIMMA package (Linear Models for Microarray Data) [24] running under the statistical language R v2.11.1. Identification of statistically significant regulation was performed using moderated t-statistic with empirical Bayes shrinkage of the standard errors [25]. Because of multiple testing, obtained P-values were corrected using Benjamini & Hochberg method to controls the false discovery rate [26]. The microarray data were deposited in the GEO online database with the accession number GSE62088 (http://www.ncbi.nlm.nih.gov/geo/query/acc.cgi?acc=GSE62088).

### Reverse Transcribed (RT)-PCR

RNA was purified from exponentially grown *B. pertussis* bacteria in virulent phase and cDNA was obtained as described in the above section. PCR was carried out using GoTaq PCR master mix (Promega) in the iCycler Thermal Cycler (Biorad Laboratories) using *recA* primers (endogenous control) (Table 3) or using primers mapping to the respective downstream region of the deleted ORFs namely *kpsT*, *kpsE* and *vipC*.

### FACS

To estimate the surface polysaccharide capsule expression, fluorescence-activated cell sorting (FACS) analysis was performed on *B. pertussis* strains grown in avirulent phase SS medium containing MgSO_4_, using the anti-Vi polyclonal mouse immune serum as described previously [17]. Each assay was performed 3 times independently.

### Intranasal infection

Specific pathogen free (SPF) female adult Balb/c mice were infected intranasally (i.n.) under anesthesia with approximately 5×10^5^ CFU of *B. pertussis* strains as described previously for lung colonization [22] and 5×10^6^ CFU of BPSM for bacterial RNA extraction. At the indicated time points, four mice per group were sacrificed (CO_2_ overdose) and their lungs were aseptically removed and homogenized. Serial dilutions of individual lung homogenates were plated and the number of CFU was determined after 4 days of incubation at 37°C. Each experiment was performed at least twice independently.

### Statistical analysis

The results were analyzed using the unpaired student *t*-test. Differences were considered significant for *p* value<0.01 and <0.05.

### Ethics statement

All the animal experiments were approved by NUS IACUC and carried out under the guidelines of the National Advisory Committee for Laboratory Animal Research (NACLAR) in the AAALAC-accredited NUS animal facilities (http://nus.edu.sg/iacuc/). All efforts were made to minimize animal suffering.

## Results

### The capsule locus expression is modulated during pertussis infection

**Figure 1:**
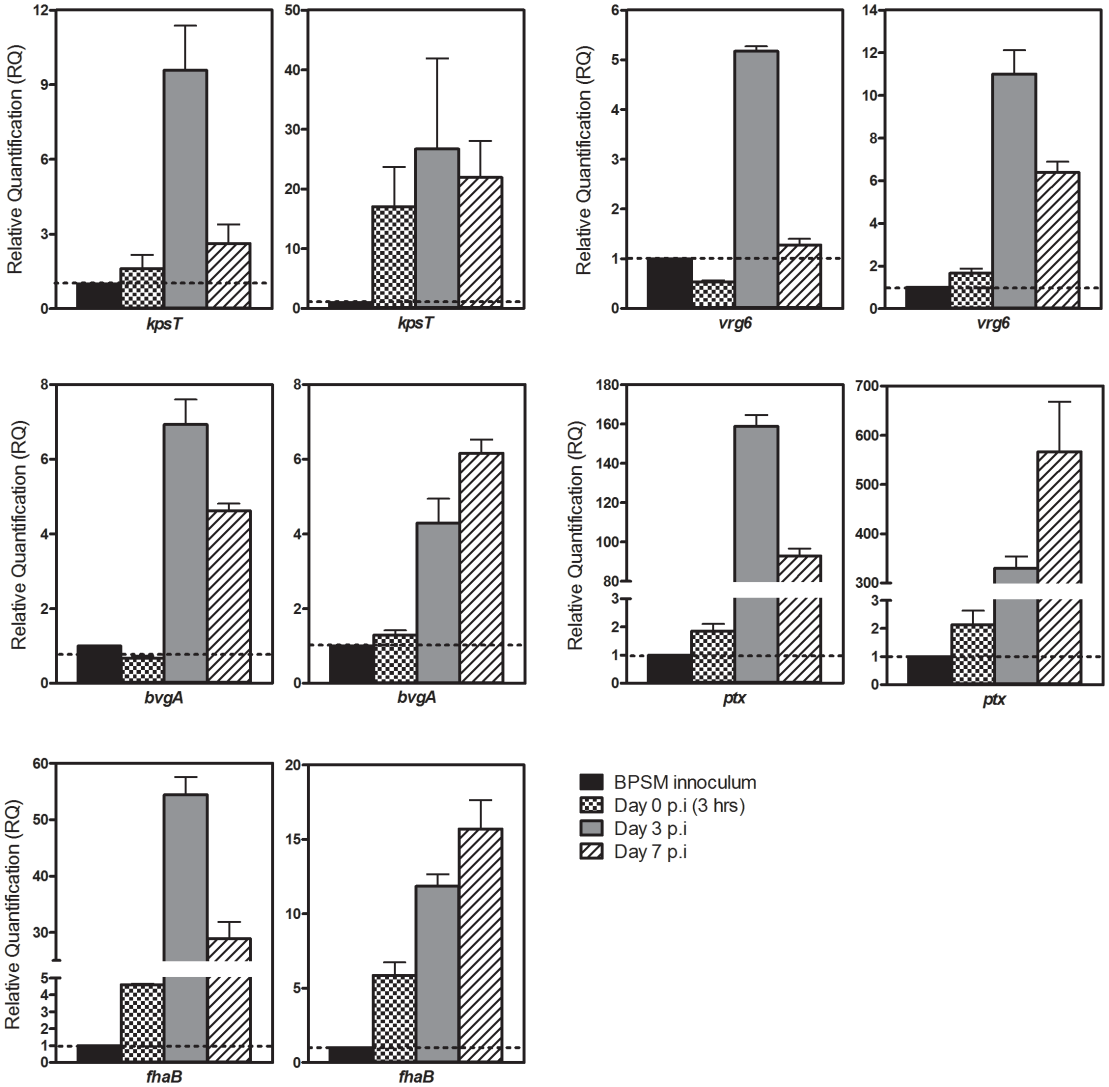
Relative transcriptional activity of *vrgs* and *vags* in BPSM bacteria recovered from mice lungs versus *in vitro* BPSM grown in virulent phase. Mice were infected intranasally with approx. 5×10^6^ CFU of BPSM and the bacteria were recovered from the mice lungs at different time points (3 hours, day 3 and day 7 p.i.). Bacterial RNA extracted and purified from group of 4 mice were pooled and subjected to real-time PCR analysis using primers mapping in the *kpsT*, *vrg6*, *bvgA*, *fhaB* and *ptx* genes. *RecA* gene was used as the endogenous control. Results are expressed as the average RQ ± SD of triplicate versus BPSM inoculum. Dotted line represents RQ equal to 1 relative to BPSM inoculum. Mock-infected mice were used as negative control. The data obtained with two biological replicates are shown. Please refer to S1 Table for Pearson correlation scores between the two independent sets.

The capsule locus in *B. pertussis* has been classified as a *vrg*, as evidenced by its optimal expression during the *in vitro* Bvg^-^ phase [14]. To investigate whether the capsule locus is expressed *in vivo*, mice were nasally infected with wild-type *B. pertussis* BPSM and at different time points p.i., the animals were sacrificed and their lungs were harvested for bacterial RNA extraction and purification. Two biological replicates were performed. Expression of the capsule locus was monitored by quantitative real-time PCR and observed to be significantly up-regulated in the mouse lungs compared to its *in vitro* Bvg^+^ expression level, with a peak of expression at day 3 p.i. (Fig. 1, S1 Table). Similar trend of expression profile was observed for another *bvg*-repressed gene, *vrg6* (Fig. 1), indicating that *B. pertussis* bacteria harvested from mouse lungs do modulate their *vrgs*' expression when exposed to host microenvironment. Similarly, the expression of *vags* including *bvgA, fhaB* and *ptx* was also found up-regulated at day 3 and day 7 p.i. compared to Day 0 (3 h p.i.) and to the *in vitro* Bvg^+^ expression level (Fig.1, S1 Table). These results thus support that *B. pertussis* modulates the expression of its capsule locus *in vivo*, thereby suggesting a role for this locus during the infection.

### Construction and characterization of *B. pertussis* capsule-deficient mutants

**Figure 2:**
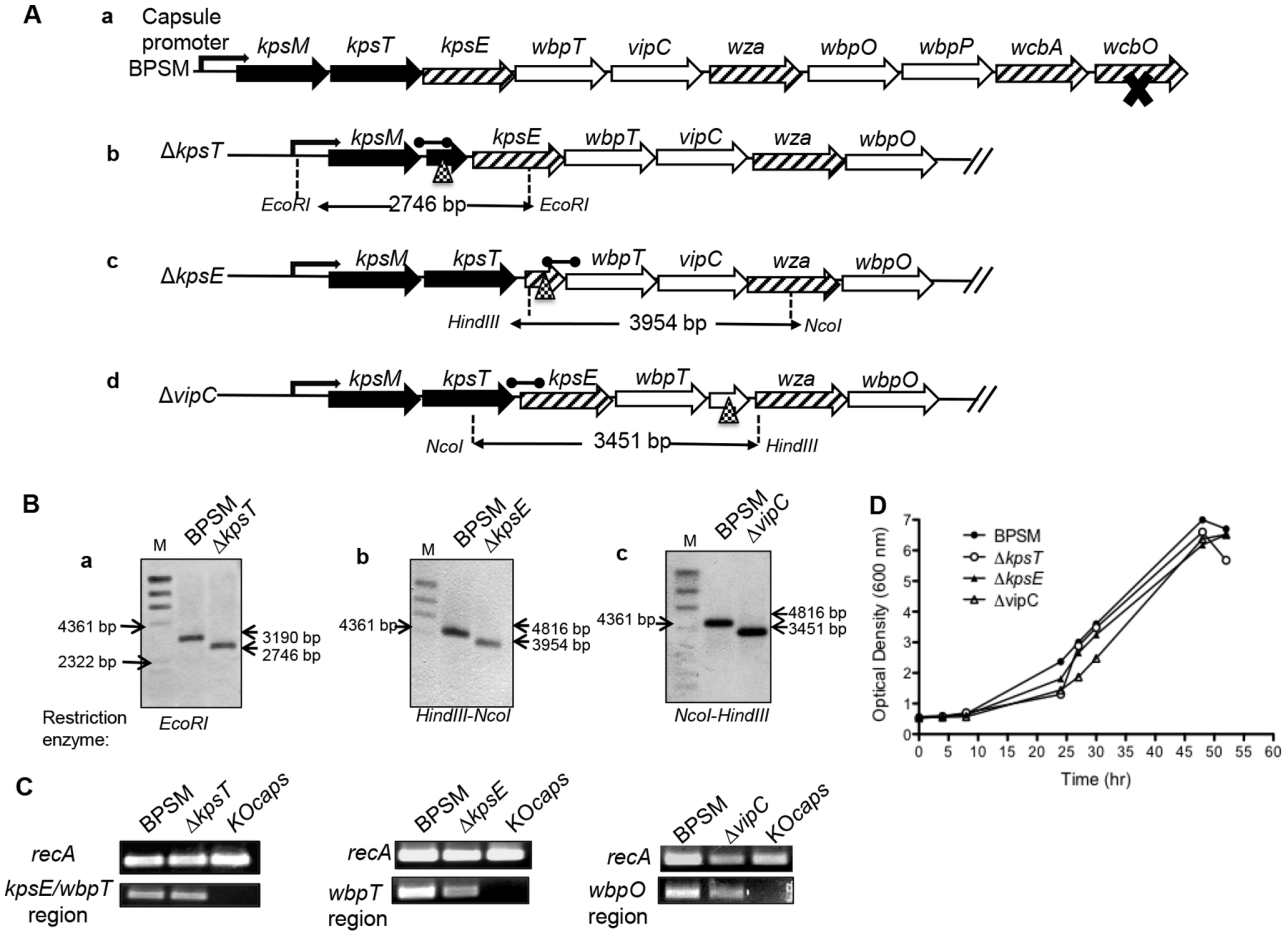
Construction of Δ*kpsT*, Δ*kpsE* and Δ*vipC B. pertussis* mutants. (**A**) **Schematic organization of** ***B*** **.** ***pertussis*** **capsule operon.** The capsule operon of *B. pertussis* BPSM strain regulated under the capsule promoter is as shown in panel **a**, black cross represents mutational insertion found in the locus. Black, hashed and open arrows represent genes involved in polysaccharide transport, polysaccharide modification/translocation and polysaccharide biosynthesis respectively. Adapted from GeneDB. Dotted triangle in panel **b** (Δ*kpsT*), **c** (Δ*kpsE*) and **d** (Δ*vipC*) indicate site of deletion that render each mutant non-capsulated. The DIG-labeled probe binding region (black rounded arrow), restriction sites and size of restriction-digested chromosomal DNA for Southern blot analysis are as shown. (**B**) **Southern blot analysis.** Restriction-digested chromosomal DNA from BPSM and Δ*kpsT*, Δ*kpsE* and Δ*vipC* were electrophoresed, transferred onto a nitrocellulose membrane and hybridized with the DIG-labeled probe (Fig. 1A for probe binding site). Panel **a**, *EcoRI*-restricted BPSM and Δ*kpsT* DNA yielded 2.7-kb and 3.2-kb respectively. Panel **b**, Hind*III*-Nco*I* restricted BPSM and Δ*kpsE* DNA yielded 4.8-kb and 3.9-kb respectively. Panel **c**, Hind*III*-Nco*I* restricted BPSM and Δ*vipC* DNA yielded 4.8-kb and 3.4-kb respectively. (**C**) **Transcription efficacy of downstream gene.** Total RNA was extracted from exponential SS liquid cultures of BPSM, KO*caps*, Δ*kpsT*, Δ*kpsE* and Δ*vipC* was reverse-transcribed followed by PCR amplification using primers specific to the endogenous control gene *recA*, and primers mapping to the respective downstream region of the deleted ORFs. The KO*caps* strain which was deleted for the entire capsule locus was used as a negative control. (**D**) **Growth kinetic profiles.** SS liquid medium was inoculated with BPSM (closed circles), Δ*kpsT* (open circles), Δ*kpsE* (closed triangles) and Δ*vipC* (open triangles) at initial OD_600 nm_ of 0.5 at time-point 0 hour. OD_600 nm_ was monitored for 52 hrs incubation at 37°C. The growth kinetics assay was performed twice independently for each strain and each culture condition. The data shown is representative of two independent experiments.

To characterize the role of the capsule locus during pertussis infection, non-polar unmarked single gene deletion mutants were constructed by targeting the capsule transport-export and biosynthesis open reading frames (ORFs) in the capsule operon, namely *kpsT*; which is predicted to encode the putative polysialic acid transport ATP binding protein, *kpsE*; the putative capsular polysaccharide export inner membrane protein and *vipC*; the capsular polysaccharide biosynthesis protein as assessed from GeneDB database. The resulting BPSM-derivative knockout mutant strains, designated as Δ*kpsT*, Δ*kpsE* and Δ*vipC* respectively, were screened by PCR and verified by Southern blot analysis (Fig. 2, A and B). Reverse transcription (RT)-PCR performed on purified total RNA from Δ*kpsT*, Δ*kpsE* and Δ*vipC* strains using primers mapping in downstream ORFs of the respective deleted regions confirmed that deletion of these individual ORFs did not lead to polar effects on the transcription of downstream genes within the capsule operon (Fig. 2 C). The KO*caps* mutant strain for which the entire capsule operon has been deleted [17] was used as negative control. Furthermore, the Δ*kpsT*, Δ*kpsE* and Δ*vipC* mutant strains displayed hemolytic and domed colony morphology on blood agar plates (data not shown) and *in vitro* growth kinetics comparable to the parental BPSM strain (Fig. 2 D), implying that these single gene deletions within the capsule locus did not affect the *in vitro* fitness of the bacteria.

### The PS capsule transport-export proteins are involved in pertussis pathogenesis

**Figure 3:**
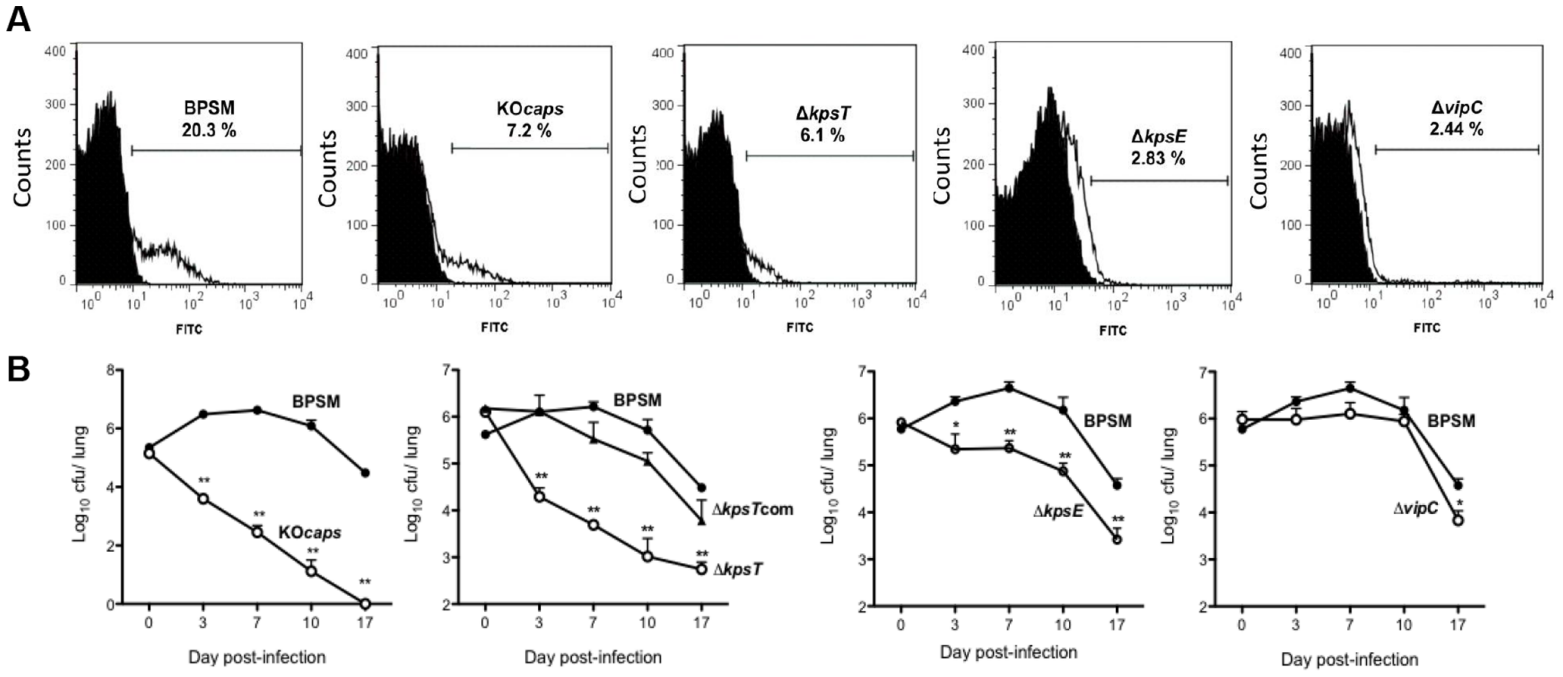
Phenotypic characterization of the Δ*kpsT*, Δ*kpsE* and Δ*vipC* mutants. (**A**) **Detection of the polysaccharide capsule at the bacterial surface.** Mouse polyclonal anti-*Salmonella typhi* Vi antigen immune serum was co-incubated with non-permeabilized BPSM, KO*caps*, Δ*kpsT*, Δ*kpsE* and Δ*vipC* bacteria strains as indicated in each flow cytometry plot. All bacteria strains were grown in Bvg^-^ phase. Anti-mouse FITC-conjugated IgG was used as secondary antibody. Isotype-matched controls are incubated with an anti-mouse antibody as shown in black histogram. The fluorescent cells were detected by flow cytometry, with 20,000 events counted for each sample. A representative experiment is shown from three independent experiments, with percentage of fluorescent cells indicated in each panel. (**B**) **Lung colonization profiles.** Balb/C mice were infected intranasally with 5×10^5^ CFU of *B. pertussis* BPSM, KO*caps*, Δ*kpsT*, Δ*kpsT*com, Δ*kpsE* and Δ*vipC* as indicated at each of graph plot. At the indicated time points, four infected mice per group were euthanized and their lungs were harvested, homogenized and plated on blood agar to determine the total number of CFU per lung. The results are expressed as the mean ± SEM of four mice per group. ** *p* value<0.01 and * *p* value<0.05 relative to BPSM. Results are representative of two independent experiments.

Deletion of *kpsT*, *kpsE* and *vipC* ORFs respectively is expected to result in the absence of the PS capsule at the bacterial surface due to the lack of effective capsule polymers transport and biosynthesis, respectively [27]. To confirm this hypothesis, FACS analysis was performed on non-permeabilized bacteria using cross-reactive anti-*S. typhi* Vi antigen immune sera as previously described [17]. Parental BPSM and capsule-deleted mutant KO*caps* [17] strains served as positive and negative controls, respectively. All the bacterial cultures were grown in Bvg^-^ phase to allow for optimal production of the PS capsule at the bacterial surface if any [17]. Due to the weak cross-reactivity of the primary antibodies detecting the loosely associated surface microcapsule, approx. 20% of the BPSM cell population gave a positive fluorescence shift, as reported previously [17]. In contrast Δ*kpsT*, Δ*kpsE* and Δ*vipC* cells consistently displayed levels of fluorescence shift comparable to that measured with KO*caps*, corresponding to nonspecific background shift (Fig. 3 A). The FACS data thus supported that in-frame deletion of the single ORF *kpsT*, *kpsE* or *vipC* within the capsule locus is sufficient to prevent the presence of the PS capsule at the bacterial surface.

Adult Balb/c mice were then nasally infected with wild type BPSM, KO*caps*, Δ*kpsT*, Δ*kpsE* and Δ*vipC*, and the bacterial loads in their lungs were monitored over time. The parental BPSM strain displayed a typical lung colonization profile with a multiplication peak at 7 days p.i. followed by a progressive clearance over the next 3 weeks p.i. (Fig. 3 B). In contrast, KO*caps* bacteria displayed no peak of multiplication, and a sharp drop in the bacterial load was observed at 3 days p.i. followed by a more progressive clearance over time (Fig. 3 B), thereby demonstrating that absence of the capsule locus affects drastically *B. pertussis* lung colonization efficacy. Expectedly, Δ*kpsT* mutant displayed a colonization profile comparable to KO*caps* (Fig. 3 B). Re-introduction of the *kpsT* ORF in the Δ*kpsT* mutant (Δ*kpsT*com strain) restored an infection profile similar to the parental BPSM strain (Fig. 3 B). Interestingly, the lung colonization profile of Δ*kpsE* was also significantly attenuated throughout the course of infection compared to BPSM albeit to a much lesser extent than the KO*caps* and Δ*kpsT* strains (Fig. 3 B). In contrast, the colonization efficiency of the Δ*vipC* mutant was comparable to the parental BPSM strain, with the exception of a lower bacterial load at day 17 p.i. (Fig. 3 B). Taken together, the data indicated that at a 5×10^5^ CFU infectious dose, the lung colonization profiles obtained with the different mutants substantially differed ranging from drastic (KO*caps* and Δ*kpsT*) to moderate (Δ*kpsE*) or no (Δ*vipC*) attenuation, although all these mutant strains lack the PS capsule at their surface. This observation thus suggests that the presence of the PS capsule at the bacterial surface is not critical for *B. pertussis* optimal colonization of mouse lungs. Our data support instead that the PS transport associated gene, *kpsT* plays a significant role in pertussis pathogenesis. Therefore, subsequent characterization in this article has focused on mechanism(s) involving the *kpsT*-mediated virulence in *B. pertussis*.

### Absence of KpsT in *B. pertussis* results in mild reduction in the production and/or secretion of major *bvg*-regulated virulence factors

**Figure 4:**
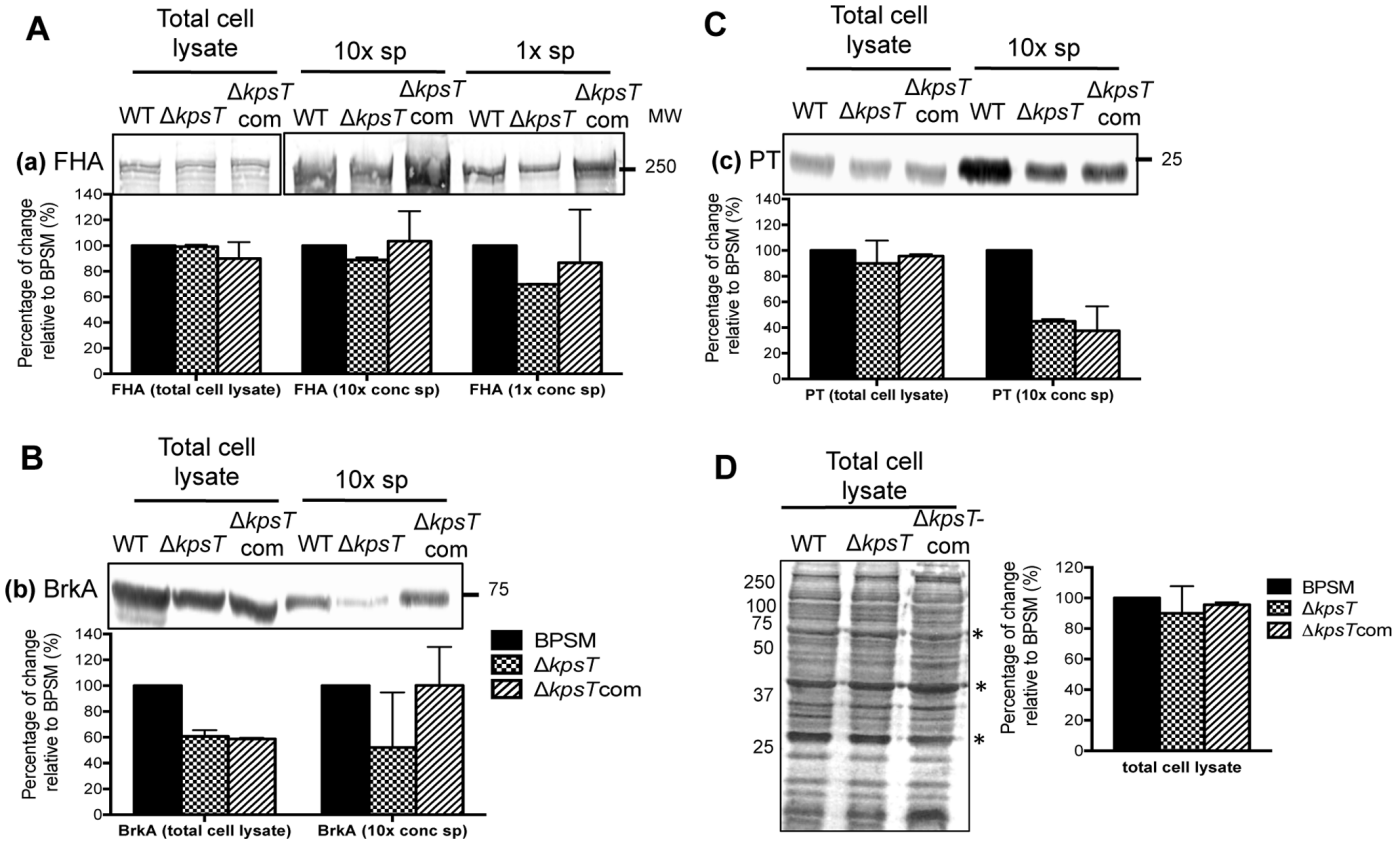
Production of *bvg*-regulated virulence proteins in Δ*kpsT* mutant. BPSM, Δ*kpsT* and Δ*kpsT*com strains were exponentially grown in virulent Bvg^+^ phase. Western blot analysis was performed on 10x concentrated or non-concentrated culture supernatants, and whole cell extracts using (**A**) anti-FHA, (**B**) anti-BrkA or (**C**) anti-PT primary antibodies. Bars at the bottom of each blot represent protein densitometry quantification expressed as percentage of change relative to parental BPSM and was determined from two independent western blot sets. Molecular weights are indicated on the right side. (**D**) SDS-PAGE and Coomassie blue staining of BPSM, Δ*kpsT* and Δ*kpsT*com whole cell lysate to estimate equal loading of protein content. Asterisks next to the Coomassie-stained bands indicate for loading control estimate. Bars represent protein densitometry quantification expressed as percentage of change relative to BPSM. Molecular weights are indicated on the left side.

We examined the production of three major virulence factors responsible for bacteria colonization including the adhesin filamentous hemagglutinin; FHA, the serum resistance protein; BrkA, and pertussis toxin; PT in Δ*kpsT* mutant compared to wild type BPSM strain all grown in Bvg^+^ phase. All bacteria cultures were harvested at mid-exponential phase (OD_600 nm_ 3). Equal loading of total protein content was confirmed by Coomassie-stained SDS-PAGE (Fig. 4D). Compared to BPSM, the band signal intensity for FHA in Δ*kpsT* 10x concentrated culture supernatant was about 30% down-regulated (Fig. 4 A). In contrast, signal intensities for FHA in the whole cell lysates appeared comparable between WT and mutant strains (Fig. 4A), indicating that secretion into the culture supernatant but not production of FHA may be compromised in Δ*kpsT*. Likewise, secretion but not production of PT seemed impaired in Δ*kpsT* (Fig. 4C). In contrast, both production and secretion of BrkA in Δ*kpsT* were visibly reduced (Fig. 4 B). Production/secretion levels of FHA, PT and BrkA were partially restored to parental level in Δ*kpsT*com strain (Fig. 4 A, B and C).

Altogether, these data demonstrated that absence of KpsT protein resulted in mild reduction of the production and/or secretion of key virulence factors in *B. pertussis*, which is unlikely to affect significantly the overall bacterial virulence and lung colonization ability observed *in vivo*.

### Absence of KpsT alters the global gene expression pattern in *B. pertussis*

**Figure 5:**
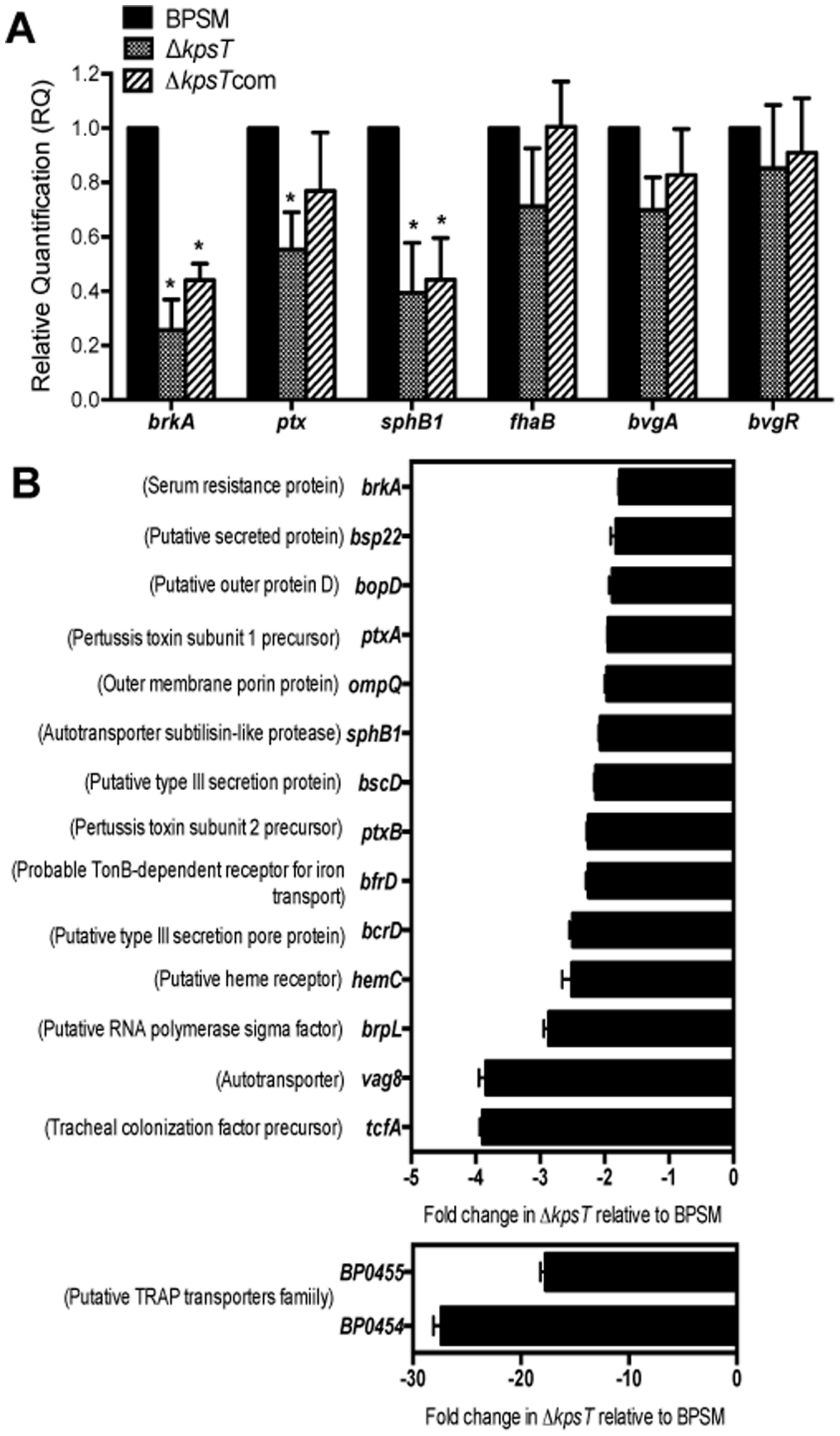
Trancriptional activity in Δ*kpsT* mutant. (**A**) **Relative transcriptional activity of** ***vags*** **.** Total RNA was extracted from BPSM (solid bars), Δ*kpsT* (dotted bars) and Δ*kpsT*com (stripped bars) strains grown in virulent Bvg^+^ phase. Real-time PCR analysis was performed using primers mapping in the *brkA*, *ptx*, *sphB1*, *fhaB*, *bvgA* and *bvgR* genes. *recA* gene was used as the endogenous control. Results are expressed as average relative quantification (RQ) vs wild type BPSM (RQ = 1). Results are representative of 3 independent experiments. (**B**) **Microarray analysis.** Total RNA was extracted from BPSM and Δ*kpsT* strains grown in virulent (Bvg^+^) phase. Microarray gene expression values were selected based on log_2_ fold change <-0.8, with adjusted *p* value<0.01. Results are expressed as average fold change Δ*kpsT* compared to BPSM, negative value indicates gene repression. Solid bars represent standard deviation of 2 independent experiments.

To gain further insights in the mechanisms responsible for the lower production and/or secretion of key virulence factors observed with the Δ*kpsT* mutant, the relative expression of the corresponding genes (*brkA, ptx*, and *fhaB*) was measured by real-time PCR. Relative quantification of these transcripts in Δ*kpsT* was compared to that obtained with wild type BPSM. Expression of the *fhaB* gene in Δ*kpsT* was not significantly different from that measured in the parental BPSM and Δ*kpsT*com strains, which is consistent with the comparable levels of expression of FHA detected by Western blot in the whole cell lysates from BPSM, Δ*kpsT* and Δ*kpsT*com (Fig. 5A). The lower levels of FHA detected by Western blot in the culture supernatant of Δ*kpsT* but not in the total cell lysate could imply that the SphB1-dependent secretion of FHA but not its production may be impaired in Δ*kpsT*. Consistently, *sphB1* transcription level in Δ*kpsT* was found to be reduced by 2.5 fold compared to the WT strain (Fig. 5A). In addition, the transcriptional activity of *brkA* and *ptx* was significantly down-regulated about 10-fold and 4-fold, respectively in Δ*kpsT* compare to BPSM (Fig. 5 A). Down-regulation of *ptx* however did not translate into lower protein levels in the whole cell lysate, although the level of secreted PT was visibly reduced in Δ*kpsT* (Fig. 4C). This discrepancy may be explained by the different sensitivity of both approaches where a limited fold change observed by real-time PCR may not be seen by western blot.

The expression level of these genes in the complemented strain Δ*kpsT*com was partially restored up 2-fold for *brkA*, 2-fold for *ptx* and 1.25-fold for *sphB1* compare to BPSM (Fig. 5 A), which correlate with the protein expression level observed in Δ*kpsT*com (Fig. 4).

Since the FHA, BrkA and PT encoding genes are regulated by the two-component system BvgA/S, we also investigated the transcriptional activity of the *bvgAS* locus [28], [29] and *bvgR* [13] in Δ*kpsT* mutant. Comparable transcriptional activities of *bvgAS* and *bvgR* were obtained in Δ*kpsT,* Δ*kpsT*com and BPSM strains (Fig. 5 A), suggesting that the lower expression of *ptx* and *brkA* in Δ*kpsT* is not directly correlated to a lower expression of the *bvgAS* locus. Together, these data indicate that deletion of the *kpsT* ORF in the capsule locus altered the expression of at least three key *bvg*-regulated genes (*brkA*, *ptx* and *sphB1*) at the transcriptional level, as well as the secretion of FHA.

To further explore the effect of *kpsT* deletion on the expression of *bvg*-regulated genes, the global transcriptional profile in the Δ*kpsT* mutant was determined and compared to its parental counterpart BPSM using DNA microarray technology which screened for a total of 3,554 *B. pertussis* ORFs. Mid-exponential Bvg^+^ phase BPSM and Δ*kpsT* cultures (OD_600 nm_ 2) were harvested and processed for RNA extraction and microarray hybridization. The global transcriptional profiling revealed a large number of genes that were significantly (adjusted *P* value<0.01) down-regulated in the Δ*kpsT* mutant (Fig. 5 B, S1 Table). The down-regulated transcripts included genes coding for autotransporters (*vag8*, *brkA*), serine protease (*sphB1*), putative RNA polymerase sigma factor (*brpL*), components and effector of the type 3 secretion system T3SS (*bcrD*, *bscD*, *bopD*, *bopN*, *bsp22*), pertussis toxin accessory genes (*ptxABDE*), tracheal colonization factor A (*tcfA*), outer membrane porin (*ompQ*) and components for iron acquisition (*hemC*, *bfrD*) (Fig. 5 B). Furthermore, consistent with our Real-time PCR analysis, expression of the *bvgAS* locus and *fhaB* was not found to be down-regulated in the Δ*kpsT* mutant (S1 Table). Notably, expression of the loci BP0454 and BP0455, which encode for the hypothetical tripartite ATP-independent periplasmic transporters (TRAP) was strongly down-modulated in Δ*kpsT* mutant (Fig. 5B and S1 Table). The energy dependent TRAP is ubiquitous in gram-negative bacteria and plays a crucial role in bacteria physiology and virulence by driving carboxylate sugar and sialic acid uptake into bacteria cell across the inner membrane [30]. Thus, in addition to support our Real-time PCR analysis, the microarray data revealed that the absence of KpsT affects negatively the expression of a large number of *bvg*-regulated genes. Such overall down-regulation is likely to be responsible for the attenuated phenotype observed with the Δ*kpsT* mutant in mice.

### Functional link between KpsT and the BvgA/S signaling pathway

**Figure 6:**
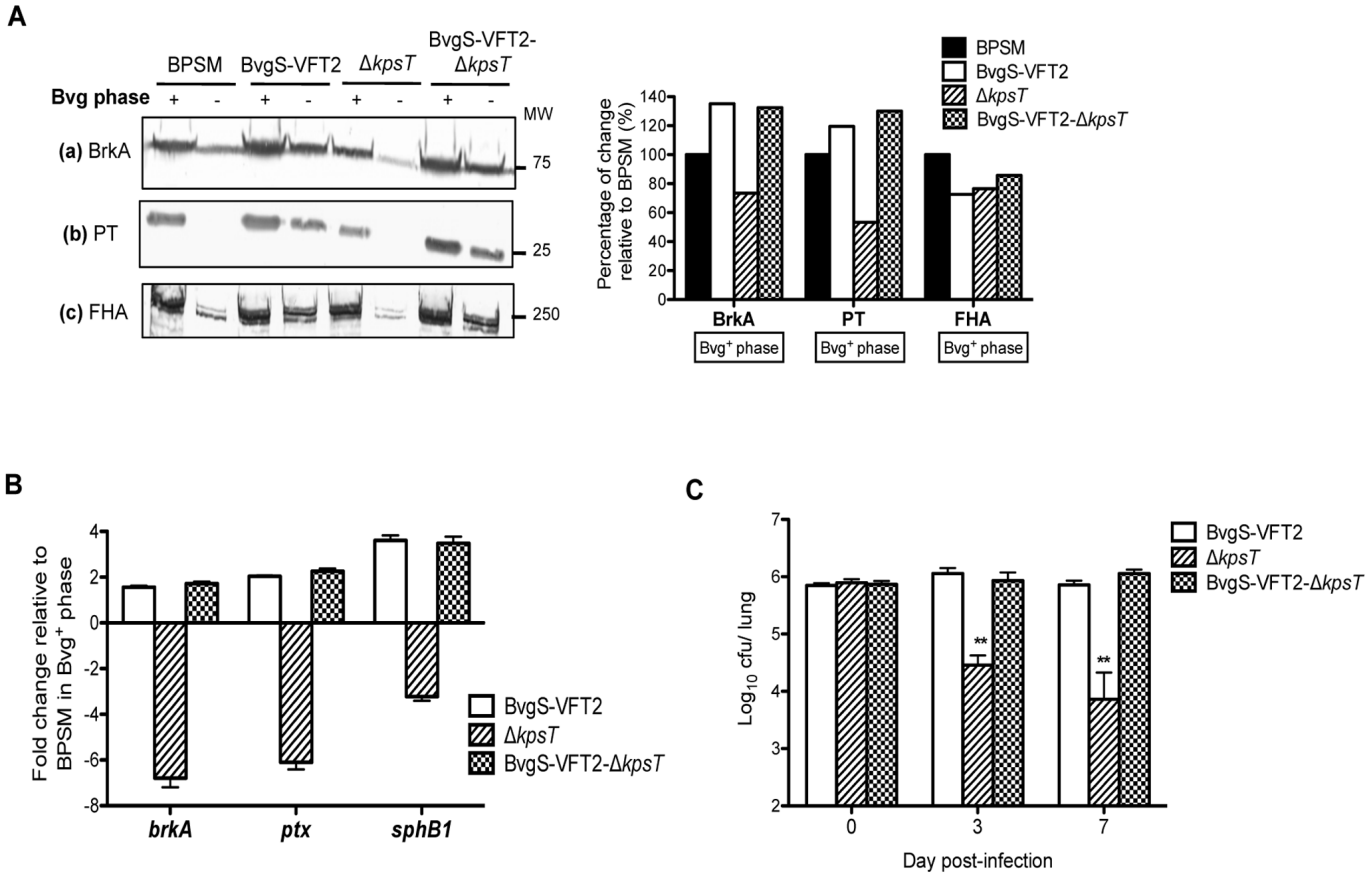
Characterization of the BvgS-VFT2-Δ*kpsT* mutant. (A) Production of *bvg*-regulated virulence proteins. BPSM, BvgS-VFT2, Δ*kpsT* and BvgS-VFT2-Δ*kpsT* strains were exponentially grown in virulent (Bvg^+^) and avirulent (Bvg^-^) phase. Western blot analysis was performed on whole cell extract (panel **a**) and 10x concentrated (panel **b**) or non-concentrated (panel **c**) culture supernatants using anti-BrkA (**a**), anti-PT (**b**) or anti-FHA (**c**) primary antibodies. Bars at the right of the blots represent protein densitometry quantification expressed as percentage of change relative to parental BPSM in Bvg^+^ phase. Results are representative of three independent experiments. Molecular weights are indicated on the right side. (**B**) **Relative transcriptional activity of** ***vags*** **.** Total RNA was extracted from BPSM, BvgS-VFT2, Δ*kpsT* and BvgS-VFT2-Δ*kpsT* strains grown in virulent Bvg^+^ phase. Real-time PCR analysis was performed using primers mapping in the *brkA*, *ptx*, and *sphB1* genes. *recA* gene was used as the endogenous control. Results are expressed for each target gene as average fold change ± SD of triplicate Ct values obtained with BvgS-VFT2, Δ*kpsT* and BvgS-VFT2-Δ*kpsT* versus the Ct value obtained with BPSM strain. The results are representative of two independent experiments. (**C**) **Lung colonization profile.** Balb/C mice were infected intranasally with 5×10^5^ CFU of *B. pertussis* BvgS-VFT2, Δ*kpsT* and BvgS-VFT2-Δ*kpsT*. At the indicated time points, four infected mice per group were euthanized and their lungs were harvested, homogenized and plated on blood agar to determine the total number of CFU per lung. The results are expressed as the mean ± SEM of four mice per group. ** *p* value<0.01 relative to BPSM. Results are representative of two independent experiments.

Our data demonstrated that *bvg*-regulated genes expression was altered in the absence of KpsT. Given the predicted localization of KpsT at the inner membrane, we hypothesized that KpsT may directly or indirectly exert its effect on the integral membrane sensor protein, BvgS sensor, thus affecting the overall BvgS-mediated signal transduction which in turn would impact on virulence gene expression levels. To test this hypothesis, we introduced the *kpsT* deletion in a BPSM-derivative Bvg^+^ phase-locked mutant [31], namely BvgS-VFT2 which contains amino acid substitutions at the periplasmic solute-binding Venus Fly Trap 2 (VFT2) domain of the BvgS sensor. BvgS-VFT2 is insensitive to environmental modulators thereby resulting in the constitutive expression of *vags* [31], [32]. We reasoned that if the effect of *kpsT* deletion on the virulence gene expression involves the BvgA/S signal transduction, a BvgS constitutive mutant may become insensitive to *kpsT* deletion. Deletion of *kpsT* ORF in the BvgS-VFT2 mutant was confirmed by PCR and Southern blot analysis (data not shown). As previously reported (34, 35), the BvgS-VFT2 mutant displayed a constitutive production of the three virulence factors BrkA, PT and FHA in non-modulating (Bvg^+^) and modulating (Bvg^-^) culture conditions (Fig. 6 A). On the contrary, production of the virulence factors was clearly down-modulated in Bvg^-^ phase for BPSM and Δ*kpsT* as anticipated (Fig. 6 A). Higher amounts of BrkA, PT and to a lesser extent FHA were detected with the BvgS-VFT2- Δ*kpsT* double mutant compared to Δ*kpsT* single mutant, with band signal intensities comparable to those observed for wild type BPSM and BvgS-VFT2 strain (Fig. 6A). Furthermore, real-time PCR analysis showed that down-regulation of the *brkA, ptx* and *sphB1* genes observed with the Δ*kpsT* single mutant was not seen with the BvgS-VFT2- Δ*kpsT* double mutant (Fig. 6B). These data thus indicated that deletion of *kpsT* in a Bvg^+^-phase locked mutant did not affect the virulence genes expression therefore suggesting that VFT2 mutation in the BvgS sensor is dominant over *kpsT* deletion. Consistently, a parental *in vivo* virulent phenotype was observed with BvgS-VFT2- Δ*kpsT* double mutant in mice (Fig. 6C). Altogether, these results indicated that deletion of *kpsT* ORF in a constitutive *bvgS* background had no effect on the expression of *bvg*-regulated genes, thus supporting a functional link between KpsT and the BvgA/S signaling pathway.

### Absence of KpsT impairs BvgS oligomerization

To further investigate the link between *kpsT* and the BvgA/S signaling pathway, a pull-down approach was undertaken. KpsT and BvgS being both located at the inner membrane, we speculated that these two proteins may physically interact. To test this hypothesis, a recombinant full-length His-tagged BvgS was expressed in *B. pertussis*. The His-tag was fused at the N-terminal end of BvgS so as not to disturb the phosphate receiver and output domains that are located at the C-terminus. The genetic construct was introduced at the chromosomal *bvgS* locus of wild type BPSM by double homologous recombination, giving rise to BPSH strain. The expression level in BPSH strain of several *bvg*-regulated virulence genes (namely *brkA*, *ptx*, *fhaB*, *bvgR* and the capsule locus) was determined by real-time PCR and was found comparable to the levels obtained with parental BPSM, thus demonstrating that presence of the His-tag at the N-terminal end of BvgS did not result in altered downstream genes expression (data not shown).

**Figure 7:**
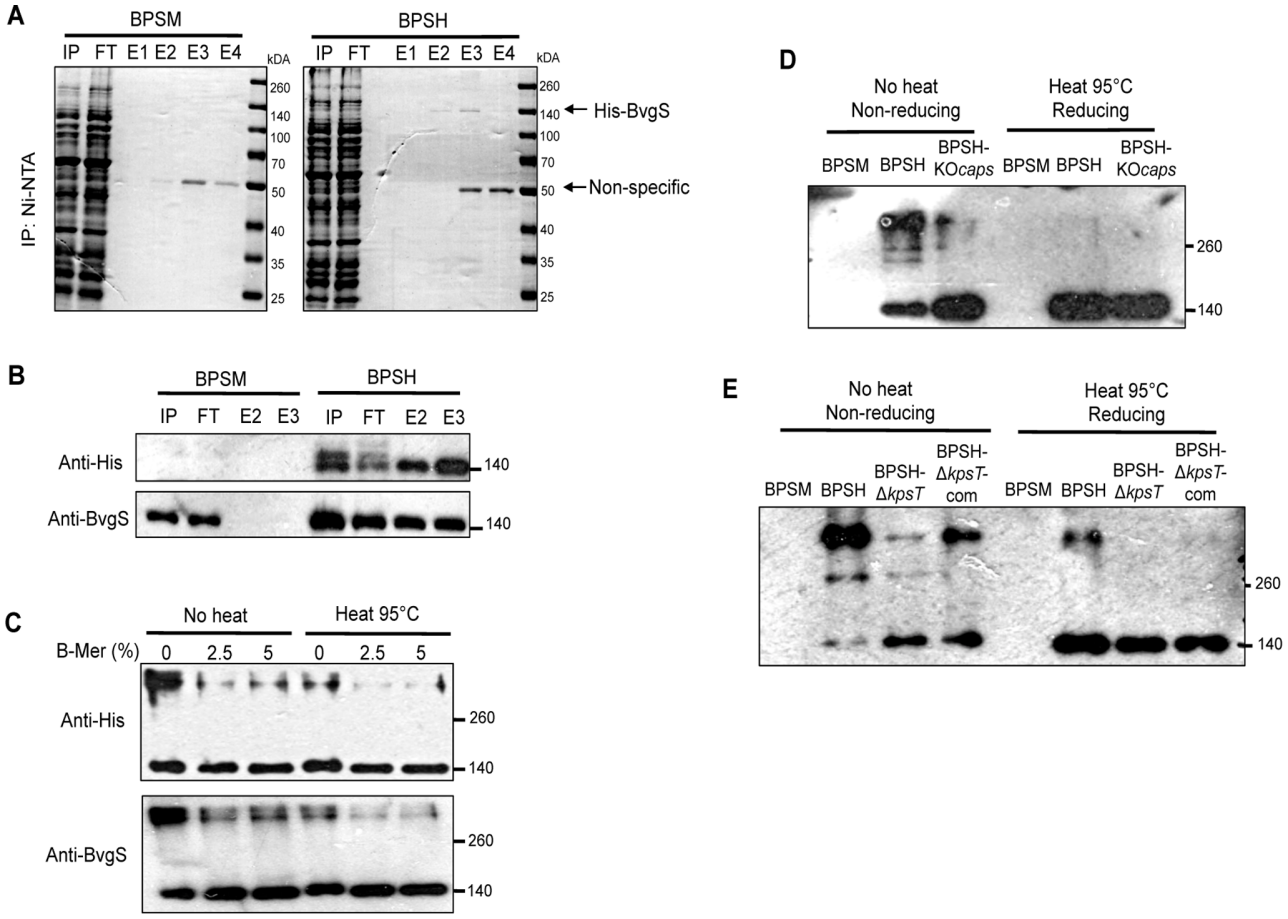
Expression and purification of His-BvgS from *B. pertussis* strains. (**A**) **Coomassie blue analysis.** 5 mg of solubilize cell lysate harvested from BPSM (untagged control) and BPSH was mixed with Ni-NTA agarose beads prior to loading onto a chromatography column. Lysate input, flow-through and batch eluted fractions were heated to 95°C for 15 min and analyzed under reducing 10% SDS-PAGE and stained with Coomassie blue. Lane IP; Input, FT; Flow through, E1; Eluted fraction 1, E2; Eluted fraction 2, E3; Eluted fraction 3, E4; Eluted fraction 4. Molecular weights are indicated on the right side. (**B**) **Western blot analysis of purified fractions with anti-His and anti-BvgS antibodies.** Lane IP; Input, FT; Flow through, E2; Eluted fraction 2, E3; Eluted fraction 3. Molecular weights are indicated on the right side. (**C**) **Detection of BvgS associated oligomers and BvgS monomer.** Purified His-BvgS from BPSH cells were mixed with equal volume of Laemmli SDS-PAGE buffer containing either no reducing agent (0% β-mercaptoethanol) or increasing concentrations (2.5% and 5% β-mercaptoethanol). The proteins samples were then subjected to either no heat or heat denaturation at 95°C for 15 min prior to SDS-PAGE analysis and Western blotted with anti-His or anti-BvgS antibody. Molecular weights are indicated on the right side. (**D**) **Detection of BvgS associated oligomers and BvgS monomer in BPSH and BPSH-KO** ***caps*** **.** Equal amount of purified His-BvgS from BPSH and BPSH-KO*caps* cells were mixed with Laemmli SDS-PAGE buffer containing either no reducing agent or with 5% β-mercaptoethanol. The proteins samples were then subjected to either no heat or heat denaturation at 95°C for 15 min. Equal amount of protein were loaded for each well for SDS-PAGE analysis and Western blotted with anti-BvgS antibody. Molecular weights are indicated on the right side. (**E**) **Detection of BvgS associated oligomers and BvgS monomer in BPSH, BPSH-Δ** ***kpsT*** **and BPSH-Δ** ***kpsT*** **com.** Equal amount of purified His-BvgS from BPSH, BPSH-Δ*kpsT* and BPSH-Δ*kpsT*com cells were mixed with Laemmli SDS-PAGE buffer containing either no reducing agent or with 5% β-mercaptoethanol. The proteins samples were then subjected to either no heat or heat denaturation at 95°C for 15 min. Equal amounts of protein were loaded for each well for SDS-PAGE analysis and Western blotted with anti-BvgS antibody. Molecular weights are indicated on the right side.

His-BvgS was successfully purified from BPSH cellular extracts under denaturing conditions, with majority of the His-BvgS protein detected in the second and third elution fractions (E2 and E3) as evidenced by the detection of a band of an apparent molecular weight (MW) of 140 kDa which corresponds to monomeric His-BvgS (predicted size of 137 kDa) from Coomassie blue stained SDS-PAGE denaturing gel (Fig. 7A). Western blot analysis using anti-His and anti-BvgS antibodies further confirmed the identity of the 140 kDa eluted protein as His-BvgS monomers in E2 and E3 from BPSH but not from BPSM extracts (Fig. 7B). A band at 50 kDa MW was also observed in E3 and E4 from both BPSM and BPSH, suggesting that this unknown protein bound to the Ni-NTA beads likely due to high His content (Fig. 7A).

To identify potential interacting partners with BvgS, BPSH cell lysates were subjected to Ni-NTA beads pull-down and purified His-BvgS fractions were exposed to increasing concentrations of reducing agent β-mercaptoethanol and heat treatment at 95°C. Western blot analysis revealed the presence of a high MW protein species (greater than 260 kDa) which reacted with anti-BvgS antibodies and disappeared in reducing conditions and/or upon heat treatment (Fig. 7C). Concomitantly, a stronger signal intensity of the 140 kDa band was observed under reducing/heat treatment conditions (Fig. 7C). This observation thus strongly suggested that BvgS is able to form high MW complexes that dissociate at high temperature and/or upon addition of β-mercaptoethanol implying that these complexes involve strong hydrophobic interactions and disulphide bonds. The high MW band (Fig. 7C) was excised from a Coomassie-blue stained non-reducing SDS-PAGE gel and subjected to Triple-TOF mass spectrometry analysis. MS analysis further confirmed with high confidence that majority of the protein complexes consisted of BvgS protein with no detection of any of the membrane proteins from the PS capsule transport machinery (data not shown). The high MW complexes captured from His-BvgS purification likely consist of BvgS multimers, confirming previous reports on the homodimerization of truncated domains of BvgS expressed in *E. coli* [33], [34]. Together these data thus suggested that the integral membrane BvgS protein does not interact directly and physically with any of the proteins form the PS capsule transport machinery.

To investigate whether proteins encoded by the capsule locus affect BvgS oligomerization, the His-BvgS construct was introduced into the KO*caps* and Δ*kpsT* mutants leading to BPSH-KO*caps* and BPSH-Δ*kpsT* strains respectively. Complemented BPSH-Δ*kpsT*com strain was also obtained. Purified His-BvgS fractions prepared from all the strains were subjected to Western blot analysis. Under non-reducing and without heat treatment, signal intensity of the high MW band>260 kDa was much lower for the BPSH-KO*caps* and BPSH-Δ*kpsT* when compared to parental BPSH (Fig. 7D & E). Instead, a higher signal intensity of the 140 kDa band was observed for both mutants compared to BPSH (Fig. 7D & E). BPSH- Δ*kpsT*com showed partial restoration of the parental BPSH phenotype (Fig. 7E). Under reducing and heat treatments, expectedly, the high MW band disappeared and comparable signal intensities of the 140 kDa band were observed for all the strains (Fig. 7D & E). Therefore, altogether these data supported that KpsT is necessary for BvgS oligomerization although no direct physical interaction seems to occur between both proteins.

### KpsT or KpsMT proteins are not sufficient to restore parental virulence in the KO*caps* mutant

Since deletion of the *kpsT* ORF did not affect the expression of the upstream and downstream genes in the capsule operon (see Fig. 2C), it is expected that the corresponding products are still produced in the Δ*kpsT* mutant. However, it is possible that in the absence of KpsT, the cellular localization and therefore function of the other membrane-associated proteins which contribute to the capsule transport machinery across the cell envelope (including KpsM, KpsE, Wza, and Wcba proteins) may be compromised. To test this hypothesis, we asked whether KpsT would be necessary and sufficient to restore a parental phenotype in the *KOcaps* mutant where the entire capsule locus has been deleted. *KpsT* ORF was thus expressed in the KO*caps* mutant, giving rise to the KO*caps*:*kpsT* strain. In addition, *kpsMT* was also expressed into the KO*caps* mutant leading to the KO*caps*:*kpsMT* strain. Indeed, in prototype *E. coli*, KpsT is a peripheral inner membrane protein that binds ATP for active transport of capsule polymers from the cytoplasm to the periplasmic face through the integral inner membrane KpsM, forming the KpsMT transporter [35], [36]. We thus reasoned that similarly in *B. pertussis*, KpsM and KpsT may form the KpsMT transporter.

**Figure 8:**
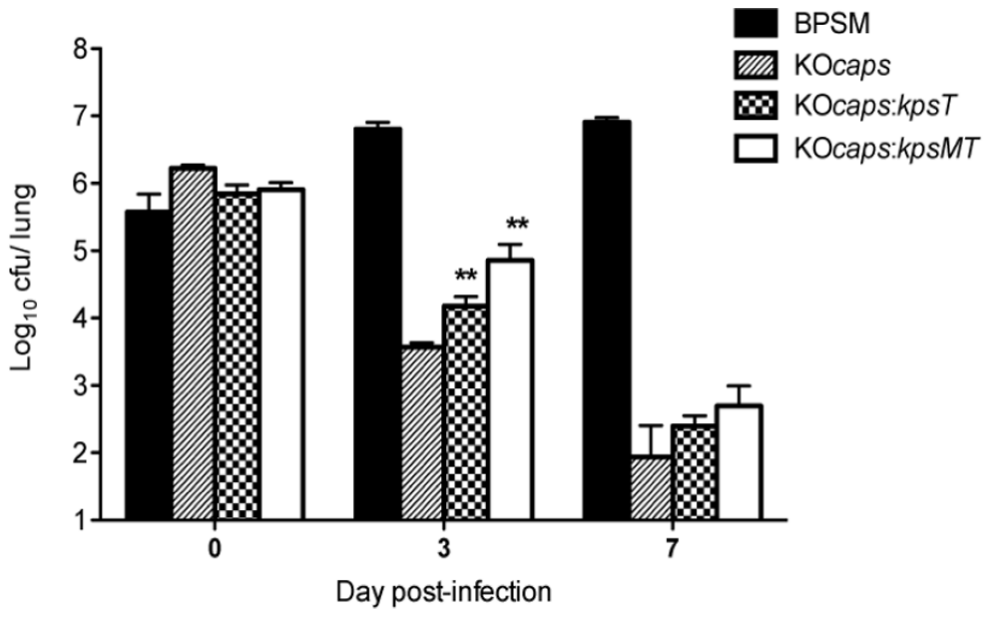
Lung colonization profile of *B. pertussis* KO*caps*:*kpsT* and KO*caps*:*kpsMT* strains. Balb/C mice were infected intranasally with 5×10^5^ CFU of *B. pertussis* BPSM (solid bars), KO*caps* (striped bars), KO*caps*:*kpsT* (dotted bars) and KO*caps*:*kpsMT* (open bars). At the indicated time points, four infected mice per group were euthanized and their lungs were harvested, homogenized and plated on blood agar to determine the total number of CFU per lung. The results are expressed as the mean ± SEM of four mice per group. ** *p* value<0.01 relative to KO*caps*. Results are representative of two independent experiments.

The lung colonization profiles of KO*caps:kpsT* and KO*caps:kpsMT* were determined in mice and compared with wild type BPSM and KO*caps* mutant. The KO*caps*, KO*caps*:*kpsT* and KO*caps*:*kpsMT* displayed a significant reduction in CFU counts at 3 days and 7 days p.i compared to the BSPM strain (Fig. 8). However, significantly higher CFU counts were obtained with KO*caps*:*kpsT* and KO*caps*:*kpsMT* at 3 days p.i compared to KO*caps* mutant alone (Fig. 8). The results here support that neither KpsT alone nor the KpsMT complex is sufficient to restore fully a parental colonization profile in *KOcaps*, This observation implies that in the Δ*kpsT* mutant, the entire polysaccharide capsule transport-export machinery across the cell wall may be structurally disrupted. Therefore, instead of KpsT alone, the entire capsule transport machinery may play a role in the BvgS-mediated signal transduction and indirectly in the overall bacterial virulence.

## Discussion

The biological role of the PS capsule, a complex structure often associated with microbial virulence and an important vaccine target for many pathogens, remains uncharacterized in *B. pertussis*. Our previous work showed that *B. pertussis* PS capsule is not involved in the classical capsule-defense mechanisms including phagocytosis, complement-mediated killing and antimicrobial peptides attack [17]. Here we provide evidence that the PS capsule transport proteins, in particular the membrane associated KpsT protein, are necessary for *B. pertussis* virulence in an indirect mode-of-action. Our data suggest a structural role for the entire PS capsule transport machinery in the cell envelope and a consequential impact on the BvgA/S-mediated signal transduction and virulence gene expression. Whilst *vrg*6 was the first *vrg* to be reported to play a role in *B. pertussis* virulence [37], later reports disputed that the attenuated phenotype observed in mice was actually due to a secondary mutation [16]. Thus, there had been no firm evidence of a possible role of a *vrg* during pertussis pathogenesis. Our work here demonstrates for the first time that a *vrg* locus (the PS capsule locus) plays a role in pertussis pathogenesis. This finding apparently contradicts a previous study which claimed that the Bvg^-^ phase is dispensable for bacterial *in vivo* virulence based on a Bvg^+^ phase locked mutant [16]. However, in that study, the authors have not checked the actual expression of the *vrgs* during *in vivo* infection with their Bvg^+^ phase locked mutant. Since *vrgs* have been shown to be modulated by BvgA/S-independent regulator RisA [38], it is thus conceivable that in the Bvg^+^ phase locked mutant *vrgs* are still expressed and modulated *in vivo*. Furthermore, during Bvg+ growth conditions, basal expression of *vrgs* can be detected and conceivably may thus play a role during infection. We have previously shown that in Bvg^+^ phase bacteria, the capsule locus is expressed at a basal level [17], [19]. Therefore, our work demonstrates for the first time that a *vrg* locus (specifically the capsule locus) i) is expressed and modulated during *in vivo* infection, and ii) plays an indirect role in *B. pertussis in vivo* virulence.

Interestingly, we observed that expression of both the vrgs *kpsT* and *vrg6*, and the vags *fhaB, ptx and bvgA* were modulated during infection. This observation thus suggests that *in vivo* a dynamic gene regulation occurs thereby implying that over time the micro-environmental conditions encountered by the bacteria vary. This is in sharp contrast with the reductionist view that *in vivo B. pertussis* bacteria are “locked” in a Bvg^+^ phase, and suggests that the *in vitro* Bvg^+^ culture conditions may not accurately represent the *in vivo* environmental conditions. Therefore, care must be taken when extrapolating data generated *in vitro* to the *in vivo* context. Indeed, the *in vitro* Bvg^–^ and Bvg^+^ phases were defined based on specific *in vitro* culture conditions in the presence or absence of modulators such as MgSO_4_ or nicotinic acid. As the true environmental signals recognized by *B. pertussis* to modulate its virulence genes expression (via the BvgA/S system) are still unknown, it is thus likely that the Bvg^–^ and Bvg^+^ states determined *in vitro* may not accurately represent the state(s) in which *B. pertussis* bacteria are *in vivo*. The more recently reported Bvg intermediate phase (Bvg^i^) supports the idea that the BvgA/S mediated modulation of the virulence genes should be seen as a rheostat instead of an on/off switch mechanism [14], [15].

The PS capsule locus in *B. pertussis* is physically distinct from the *bps* locus (BP1942-BP1944) which encodes proteins involved in biofilm formation. Biofilm formation has recently been reported in the Bordetella genus [39]–[41]. Impairment in biofilm production in both *B. bronchiseptica* and *B. pertussis* due to deletion within the *bps* locus resulted in a reduced bacterial adherence to the murine nasal cavity and trachea whereas the colonization of murine lungs was not affected [42], [43]. It was also reported that expression of the *bps* locus is not under the regulation of the BvgA/S two-component system [44]–[46]. Therefore, the biofilm structure observed in *B. bronchisptica* and *B. pertussis* is independent from the PS capsule resulting from the *kpsT* genetic locus expression.

The arrangement of the 10-kb capsule operon in *B. pertussis* is similar to that of *E. coli*, *N. meningitides*, *S. typhi* and *H. influenzae* type b encoding the PS capsule ABC-transporter dependent system [44]. KpsMT is part of the putative capsular PS transport system in *B. pertussis.* The predicted amino acid sequence of *B. pertussis* KpsT protein exhibits significant degree of homology with several other proteins responsible for active transport of capsular polysialic acid polymers, including KpsT from *E. coli* (40% identity) and HexA from *Pasteurella multocida* (44% identity), strongly supporting that *B. pertussis* KpsT performs a similar function [44]. According to PSORTb signal peptide prediction program and ExPASy TMPred trans-membrane topology prediction software [47], the *B. pertussis* KpsT protein does not contain a conserved signal peptide and displays a small transmembrane segment (AA 74-93), probably a lipid anchor, respectively (data not shown). Based on homology with the KpsT/KpsM transporter unit described in *E. coli*, it is speculated that similarly in *B. pertussis*, KpsT dimers are localized at the inner membrane periphery facing the cytoplasm and interact with KpsM dimers which are fully inserted into the lipid bilayer with 5–6 predicted transmembrane domains. In Δ*kpsT* mutant, a truncated 100 amino acid-long KpsT protein made of the first 65 Nt AA and the last 35 Ct AA is expected to be produced. It could be argued that this truncated protein may still be able to insert in the plasma membrane thereby interfering with the membrane integrity and be responsible for the attenuated phenotype seen with Δ*kpsT*. However, the truncated KpsT protein does not contain the predicted lipid anchor region and is therefore unlikely to insert in the plasma membrane. It is also unlikely that the truncated KpsT protein has retained its ability to fold into native conformation, dimerize and interact with KpsM dimers. Thus, the truncated 100 AA-long KpsT protein produced in Δ*kpsT* mutant is unlikely to destabilize the plasma membrane integrity and cause some toxicity.

Nevertheless, deletion of *kpsT* in *E. coli* was shown to lead to accumulation of PS polymers at the inner cell periphery due to defect in the PS capsule polymer trafficking process [27], [36]. Similarly, intracellular PS polymers accumulation may also occur in *B. pertussis* Δ*kpsT*, and this may affect the cell viability and overall fitness. However, no significant *in vitro* growth defect was noticed for this mutant. In addition, the KO*caps* mutant deleted for the entire capsule locus and for which PS polymers accumulation does not occur, displayed an attenuation profile *in vivo* that was comparable to that seen with the Δ*kpsT* mutant. These observations therefore do not support a role for intracellularly accumulated capsular PS polymers in the Δ*kpsT in vivo* attenuated phenotype.

Our data indicated that absence of the membrane-associated protein KpsT (and to a lesser extent KpsE) affects significantly pertussis pathogenesis. The varying degrees of attenuation of the lung colonization profiles seen with the three acapsulated Δ*kpsT*, Δ*kpsE* and Δ*vipC* mutants implied that instead of the PS capsule itself displayed at the bacterial surface, the membrane-associated protein KpsT and to a lesser extent KpsE, play a role in pertussis pathogenesis. Genome wide microarray analysis in Δ*kpsT* mutant revealed significantly reduced transcriptional activity of a variety of genes that are *bvg*-regulated or not, and among which a number encode virulence factors associated with bacterial colonization. These findings thus suggested that the membrane associated KpsT protein participates to a global gene regulation mechanism. Real-time PCR analysis on key *bvg*-regulated genes validated the microarray data. The differential down-regulation observed with *bvg*-regulated genes in Δ*kpsT* likely reflects the different affinities of each promoter for phosphorylated BvgA (P-BvgA), the transcriptional regulator of the BvgA/S two component system [48]. As such, a decrease in P-BvgA levels will first affect the *bvg*-regulated promoters with low affinities for P-BvgA [49]. Thus absence of the KpsT protein in *B. pertussis* resulted in the down-regulation of a large number of virulence genes involved in the colonization efficacy which is likely responsible for the attenuated phenotype seen with Δ*kpsT* in mice. Consistently, previous work has shown that, whereas the absence of a single virulence factor in *B. pertussis* resulted only in mild or no attenuation, multiple deletions in genes encoding adhesins and toxins significantly impaired the ability to colonize the mice lungs, supporting some degree of functional redundancy among the different virulence factors [50], [51].

Partial restoration only of the parental virulence gene expression and protein production was observed with the complemented strain Δ*kpsT*com. We speculate that the expression of *kpsT* ORF on a replicative plasmid in this strain may lead to suboptimal ratio of the different PS capsule associated proteins in the cell wall, which may possibly affect BvgS-mediated signal transduction and downstream gene expression. Nevertheless this partial restoration at the transcriptional and protein levels seems to be sufficient to fully restore a parental phenotype *in vivo.*

Deletion of *kpsT* in a Bvg^+^ phase-locked background did not lead to reduced *vag* expression and impairment of *in vivo* virulence as observed with the parental Δ*kpsT* mutant, thus suggesting a regulatory link between KpsT and the BvgA/S-mediated signal transduction pathway. The model of a two-component system comprises of a sensor kinase and response regulator, which is often thought to work according to a linear mode of action, from the perception of stimulus to downstream phosphorelay activation and transcriptional responses. However, many studies have now shown that the complexity of a bacterial two-component system was generally overlooked. Direct and/or indirect cross-talks between two-component systems and non-cognate partners have been widely reported as a new paradigm in bacterial signal transduction [52]–[57]. They affect the downstream phosphorylation activity of the sensor and response regulators, thus modulating the overall output of the two-component system [57]. Furthermore, and more relevant to our own observations, reports of inner membrane proteins interacting physically and influencing a two-component sensor kinase activity have been recently described in different pathogens [53], [58], [59]. We thus hypothesized that KpsT may interact with BvgS sensor thereby affecting its signal transduction activity. However, our pull-down assays supported that BvgS molecules are unlikely to interact physically with neither KpsT nor any other proteins from the PS transport complex, although additional experiments would be necessary in order to confirm this absence of direct physical interaction. Instead, we showed that absence of KpsT impaired the ability of BvgS to form high MW oligomers. It is to be noted that despite the presence of guanidine hydrocholoride during the purification process, we were still able to observe high MW BvgS complexes. However, large hydrophobic proteins, in particular membrane proteins or proteins that contain proline rich homeodomains have been reported to be resistant to denaturation by urea or guanidine hydrochloride [60]–[62]. It has been suggested that in eukaryotic system, proline rich regions mediate protein dimerization and oligomerization [62], [63]. BvgS sensor contains two separate alanine-proline rich regions within the cytoplasmic histidine kinase and receiver domains [12], [64]. Whether these features are responsible for maintaining the conformational tension between BvgS oligomers in *B. pertussis* remain to be investigated. In the context of chemical bonds within BvgS oligomers interface, it is also plausible that strong covalent bond and disulphide bonds exist within the distinct macromolecular structure associated with BvgS.

Our pull-down data demonstrate that absence of *kpsT* or the entire capsule locus affects oligomerization, presumably homodimerization, of the BvgS sensor. Biochemical and structural evidences have indeed confirmed that BvgS forms homodimers at two domains within the C-terminal cytoplasmic region, namely the transmitter and receiver-output domains [34], as well as at a specific cytoplasmic sensory domain, known as PAS domain [65]. Moreover, active phosphotransfer could be reconstituted in *trans* between BvgS domains, thus further supporting the dimerization capacity of BvgS *in vivo* [33], [34]. Dimerization and higher order oligomerization of signaling complexes in general and of BvgA/S in particular are believed to influence conformational and mechanical stability necessary for intrinsic phospho-transfer activity and activation of downstream regulator proteins [65]–[67]. Mutational studies on BvgS PAS domain abolished BvgS dimer stability and resulted in functional consequences of BvgS signal transduction from the periplasm to cytoplasmic domain [65]. Therefore, our data indicated a role for KpsT in BvgS oligomerization, thereby impacting on signal transduction and downstream gene activation. Fine tuning of virulence gene expression during *B. pertussis in vivo* infection is crucial for bacterial virulence during different stages of infection within the host environment [68]–[70].

The precise mechanisms underlying the role of KpsT in BvgS oligomerization are yet to be fully deciphered, and since KpsT does not physically interact with BvgS, we reasoned that it may have an indirect effect via its role on the overall membrane structure and integrity. We propose that absence of KpsT, the putative ATPase cognate partner of the integral membrane KpsM, may eventually lead to a complete disorganization of the entire capsule transport machinery within the cell envelope which may affect the overall membrane integrity and permeability. This hypothesis is supported by the observation that i) *in vivo* attenuation was also seen with a Δ*kpsE* mutant, and ii) re-introduction of KpsT or KpsMT into the KO*caps* mutant was not sufficient to restore a parental phenotype, thus supporting that absence of KpsT in the Δ*kpsT* mutant may actually affect the trans-envelope complex formed by the PS capsule transport proteins. Further structural analysis would be necessary to test this hypothesis.

Alternatively or in addition, absence of KpsM/T ABC transporter in Δ*kpsT* mutant may affect the asymmetric lipid distribution in the outer membrane (OM) thereby resulting in increased permeability to chemical agents and impairing detection of environmental stimuli. Such paradigm was reported in *E. coli* where mutation of an ABC transport system was found to alter phospholipids (PL) accumulation in the outer leaflet of the OM [71]. This ABC transporter belongs to the Mla pathway, a bacterial intermembrane PL trafficking system which is composed of at least six proteins with one component in each cellular compartment. Similarly, KpsM/T together with the other PS capsule transport proteins may also play a role in PL distribution at the OM of *B. pertussis* bacteria, although this hypothesis is purely speculative.

In conclusion, our findings led to a novel finding that the *B. pertussis* PS capsule transporter-export machinery and in particular KpsT are necessary for optimal expression of virulence genes and therefore play an important role in pertussis pathogenesis. Mechanistically, we propose that KpsT and likely the capsular transporter-exporter complexes participate to the plasma membrane structure and integrity, which are crucial for the conformational integrity and optimal functionality of membrane proteins such as BvgS sensor.

## Supporting Information

S1 Table **Pearson correlation between two biological replicates of real-time PCR data sets.** The gene name, time-points p.i, and fold change/relative quantification (RQ) values of technical triplicates are indicated for both biological replicates performed as described in Fig. 1. Pearson correlations of the RQ values across the 3 time-points between the 2 independent datasets are shown. All of the genes showed positive correlation values between 1 to 0.5 and positive regulation (RQ value>1) for all time-points between the 2 datasets.
(DOCX)
