# Supplementary material for: Evidence for a Role of the Polysaccharide Capsule Transport Proteins in Pertussis Pathogenesis
Source: PLoS One. 2014 Dec 12;9(12):e115243. doi: 10.1371/journal.pone.0115243 (PMC4264864; doi:10.1371/journal.pone.0115243)
Supplement: S1 Table — Pearson correlation between two biological replicates of real-time PCR data sets. The gene name, time-points p.i, and fold change/relative quantification (RQ) values of technical triplicates are indicated for both biological replicates performed as described in Fig. 1. Pearson correlations of the RQ values across the 3 time-points between the 2 independent datasets are shown. All of the genes showed positive correlation values between 1 to 0.5 and positive regulation (RQ value>1) for all time-points between the 2 datasets. (DOCX) [file pone.0115243.s001.docx]

**Supplemental information**

**Table S1. Pearson correlation between two biological replicates of real-time PCR data sets.**

The gene name, time-points p.i, and fold change/relative quantification (RQ) values of technical triplicates are indicated for both biological replicates performed as described in Figure 1. Pearson correlations of the RQ values across the 3 time-points between the 2 independent datasets are shown. All of the genes showed positive correlation values between 1 to 0.5 and positive regulation (RQ value>1) for all time-points between the 2 datasets.
